# Barriers to healthy eating by National Health Service (NHS) hospital doctors in the hospital setting: results of a cross-sectional survey

**DOI:** 10.1186/1756-0500-1-69

**Published:** 2008-08-28

**Authors:** James Winston, Carol Johnson, Sue Wilson

**Affiliations:** 1 Department of Primary Care and General Practice, University of Birmingham, Edgbaston, Birmingham, B15 2TT, UK

## Abstract

**Background:**

With high levels of obesity and related illness, improving the health of the nation is a major public health concern. This study aimed to identify factors that prevent healthy eating among doctors, and that are associated with satisfaction with catering services.

**Findings:**

*Methods: *Cross-sectional survey of 328 NHS doctors working in two NHS Trusts with on-site hospital canteen. Questionnaire to establish perceived barriers to healthy eating, weekly use and satisfaction with the hospital canteen, lifestyle and dietary habits, gender, age, height, weight, job details, and affect.

*Results: *70% of doctors reported using their hospital canteen each week, with 2 visits per week on average.

Canteen opening times, lack of selection and lack of breaks were the most commonly perceived barriers to healthy eating. Availability of healthy options caused the most dissatisfaction. Only 12% felt the NHS was supportive of healthy eating. 74% did not feel their canteen advocated healthy eating. Canteen use is associated with younger age (r = -0.254, p < 0.0001) and health score (r = 0.123, p = 0.049).

**Conclusion:**

Interventions to encourage regular meal breaks, eating breakfast and drinking more water each day need developing. Improved canteen accessibility and availability of healthy options at evenings and weekends may be beneficial.

## Background

One-quarter to one-third of all ill health in the world today may be attributed to environmental factors, particularly poor diet and smoking [1]. Poor diet and obesity are associated with diet related illnesses such as heart disease and diabetes and diet has an influence on concentration, memory and attention span [2,3], motor performance [4], mood [5], and tiredness [2,4]. Tiredness is associated with cognitive and motor impairments, injuries, and mistakes [6,7]. Optimal performance during demanding mental or physical activities requires adequate nutritional input [2-4,6,7]. With long working hours, emotional stresses and high work load, medicine is an example of a mentally and physically demanding activity requiring high levels of cognitive and motor performance [7,8].

The only previous survey of catering provision and barriers to healthy eating in health care professionals was undertaken among a small group of nurses across a limited number of specialties [9,10].

Health promotion is a multifactorial process operating on individuals and communities, through education, prevention and protective measures [11]. *Choosing Health *[12] is the British Government's guide to improving the nations' health through health promotion. Illness and absenteeism among health care workers may lead to reduced patient care and increased work and stress for other team members [13]. This concept is also referred to as the "*Health Promoting Hospital*" [11].

As one of Britain's biggest employers, health promotion within the NHS could potentially influence the health of many [14]. Hospitals can also promote health in their community by acting as a "change agent" through displaying clear support for health promotion [11,12]. Applying these concepts to the hospital canteen suggests that canteens serving healthy options could lead by example and promote health by advocating healthy eating.

A number of Government strategies have aimed to improve the working environment for NHS employees [14,15], but have not addressed catering facilities or nutritional needs. Assessing staff requirements, and satisfaction with catering facilities, may be associated with retention and recruitment [14,15]. Although improvements to doctors' working lives have been made through the European Working Time Directive [16] and initiatives to make the NHS a smoke-free workplace [12], nutritional and dietary needs have not been addressed.

## Findings

### Aim

To identify areas of a doctor's working environment that prevent healthy eating, describe doctors' satisfaction with canteen services, and establish the extent to which the canteen is used by doctors. Associations between doctors' health behaviours, affect, perceived barriers to healthy eating, and canteen use were also examined.

### Methods

A cross sectional survey was conducted of hospital doctors with access to a hospital canteen in two NHS Trusts. [See Additional File 1]

The South Birmingham Student Ethics Committee granted ethical approval (Reference S/2006/008), R&D approval was attained in each participating Trust. UHB reference RRK3018, BSMHT reference 775.

### Results

**Figure 1:**
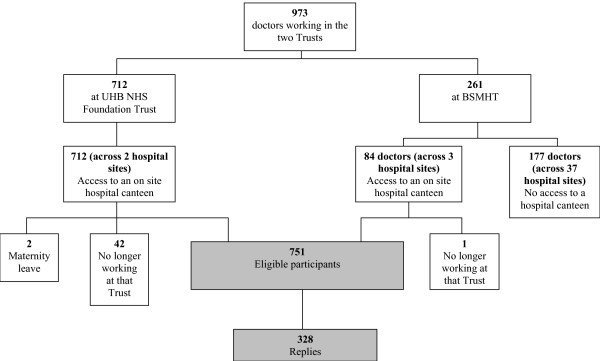
Consort Diagram.

The eligible sample comprised 751 doctors (Figure 1) from two large teaching hospitals, one general psychiatric hospital, and two forensic psychiatric hospitals. The overall response rate was 43.7% (328/751); and varied by Trust (77% (64/83) at BSMHT and 37% (246/668) at UHB) and job grades (Table 1).

**Table 1:** Response rate by job grade

**Job Grade**	**Eligible** **participants (n)**	**Replies ** **Received**	**Response ** **Rate (%)**
Foundation Year 1 (FY1)	47	24	51.1
Senior House Officer (SHO)	163	49	30.1
Specialist Registrar (SpR)	212	68	32.1
Associate Specialist	11	9	81.8
Staff Grade	17	11	64.7
Consultant	296	151	51.0
Dental/Medical Practitioner	15	0	0
Hospital Practitioner	3	0	0
Not specified	32	16	50

**Total**	**751**	**328**	**43.7**

Left the trust/On maternity leave	45		

### Perceived Barriers

**Figure 2:**
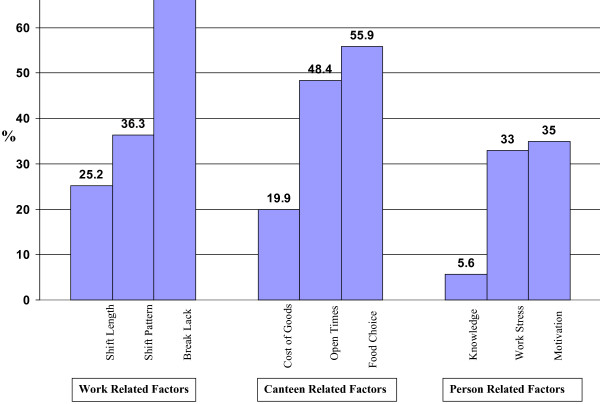
Factors that prevent health Eating in Doctors (n = 306).

Only 12% (n = 37/310) of respondents reported their employer (the NHS) was supportive, 35% (n = 109/310) thought their employer was unsupportive of healthy eating, and 53% (n = 164/310) were undecided. The average number of barriers to healthy eating identified by each doctor was 3.3 (range 0–9, SD 1.8), with lack of breaks (66%, n = 203/306, range between the five hospital sites: 20.0%–70.7%), lack of selection (56%, n = 171/306, range between hospital sites: 40%–100%), and canteen opening times (48%, n = 148/306, between the sites: 37.5%–60.0%) being the three most commonly identified barriers (Figure 2). Less than half the doctors (47%, n = 149/315, between hospital sites: 20%–57.9%) reported taking regular meal breaks.

### Canteen Satisfaction

The mean canteen satisfaction score was 8.6 (range 0–18, SD 2.98, n = 262). Only 39 (14.9%) doctors had a satisfaction score of 12 or more, with 62 (23.7%) having a score of 6 or less. Satisfaction scores varied by hospital site; mean satisfaction scores for each site ranging from 7.7 (n = 110, SD = 3.0) to 10.9 (n = 40, SD = 2.78) (one-way anova, df = 4, F = 9.98, P < 0.0001). 83% (n = 259/311) of doctors reported nutrition and healthy eating to be important factors influencing their work performance. However, 74% (n = 200/269) of doctors did not feel their canteen advocated healthy eating. Availability of healthy options caused the most dissatisfaction among respondents (Table 2), with 46% (n = 121/262) not completely satisfied and 26% (n = 67/262) not at all satisfied. 39% (n = 90/234) of doctors felt the provision of healthy food changed over the course of the day, with poor or absent evening and weekend catering provision being the most common reason given.

**Table 2:** Doctor Satisfaction with Aspects of Canteen Services (row percentages in brackets)

	Very satisfied	Satisfied	Not completely satisfied	Not at all satisfied	N
Cost of goods	19 (7)	132 (50)	85 (32)	28 (11)	264
Location of canteen	53 (20)	166 (62)	32 (12)	16 (6)	267
Opening times	14 (5)	84 (32)	121 (46)	44 (17)	263
Selection of food and drink	7 (3)	79 (30)	132 (50)	44 (17)	262
Availability of healthy options	4 (1.5)	70 (26.5)	121 (46)	67 (26)	262
Appearance/environment of the canteen	13 (5)	160 (61)	57 (22)	32 (12)	262

Only 10% (n = 28/290) of doctors had access to a staff-only canteen, yet 77% (n = 174/226) reported preferring staff-only facilities. 76% (n = 29/38) of vegetarians felt the canteen did not cater for their dietary needs.

### Canteen Use

70% (n = 229/328) of doctors reported using their hospital canteen each week, with on average 2.07 (SD 2.09, range 0–10) visits per week on average (see Additional file 2). 67% (n = 219/328) of doctors purchased food or drink from the canteen; main meals (44%, n = 145/327) and sandwiches (41%, n = 134/327) being the most common purchases. There was a negative association between canteen use and age (r = -0.254, p < 0.0001, n = 313). One-way between-groups analysis of variance (ANOVA) revealed a difference in canteen use with age and job grade (see Additional file 2 and see Additional file 3). Higher mean canteen use was observed by Foundation Year 1's (FY1) and Senior House Officers (SHO) than by consultants (p = 0.001; Eta squared was 0.088 (medium to large effect size) and among doctors aged less than 35 compared to doctors aged 35 to 45 (p = 0.002) and greater than 45 (p < 0.0001), Eta squared was 0.056 (medium effect size). (see Additional file 2).

### Health behaviours

77% (n = 238/309) of doctors considered themselves healthy eaters, and on average rated their health on that day as 72 (SD 16.8, range 0–100, n = 308). Mean health score was 6.9 (SD 2.7, range 0–15, n = 257) and no significant differences were observed between age groups, job grades or genders (Table 3). Individual dietary behaviours are summarised in Table 4. The average number of glasses of water consumed daily was 3.1 (SD 2.4, range 0–12, n = 314), with only 14% drinking the recommended six to eight glasses per day.[17] The average number of separate episodes of aerobic exercise per week was 2.2 (SD 1.8, range 0–8, n = 315). There was weak positive correlation between health score and canteen use (r = 0.123, p = 0.049, n = 257). Satisfaction score, age, BMI, gender, and job grade were not associated with health score.

**Table 3:** Health Behaviour Score According to Age, Job Grade and Gender

Category		Mean Health Score	N	Standard Deviation	Range	Minimum Health Score	Maximum Health Score
Age	<35	7.28	105	2.56	13	2	15
	35–45	6.58	77	2.98	14	0	14
	>45	6.62	74	2.49	11	2	13

Job Grade	FY1	7.15	20	2.60	12	2	14
	SHO	7.92	39	2.56	12	3	15
	SpR	6.29	59	2.22	11	1	12
	Associate Specialist	6.00	8	3.74	9	1	10
	Staff Grade	7.56	9	2.74	9	4	13
	Consultant	6.85	117	2.81	14	0	14

Gender	Male	6.95	153	2.71	13	1	
	Female	6.79	102	2.66	15	0	14

**Table 4:** Summary of Dietary Behaviours (n = 328)

**Behaviour**	**N**	**%**
Eat breakfast		
Every/most days	232	70.8
Once/twice a week	38	11.6
<Once a week/never	46	14.0

What eaten for breakfast		
Brown toast	211	64.3
White toast	61	18.6
Chocolate	4	1.2

Snacking		
<Once a day	195	59.5
Once a day	103	31.4
Regularly, throughout the day	18	5.5

Fruit & vegetables (per day)		
5 or more	85	25.9
2 or more, but < 5	180	54.9
< 2	49	14.9

Glasses of water (per day)		
6 or more	46	14.0
3 or more, but < 6	109	33.2
< 3	159	48.5

What do you drink when you are thirsty?		
Water or fruit juice	198	60.4
A fizzy drink	98	29.9
Sugar free drink	18	5.5

Salt use		
Eat low salt foods	90	27.4
Use salt only for cooking	198	60.4
Add salt to all meals	26	7.9

Take away meals		
Once a month or less	160	48.8
Once a week	138	42.1
> Once a week	18	5.5

**Note**: where there are missing responses, percentages do not total to 100%.

### Affect

Mean positive affect score was 29.6 (SD 7.29, range 10–48, n = 291) and mean negative affect score was 12.7 (SD 3.33, range 10–27, n = 291). Both positive and negative affect scores were lower than reported norms for the UK adult population [18]. There were differences (p < 0.0001) in positive affect between males (Mean 30.9, SD 7.1) and females (Mean 27.7, SD 7.2). Positive affect was also weakly associated with age (r = 0.136, p = 0.021, n = 289).

## Discussion

This study demonstrates that many doctors felt their employer and work environment were unsupportive of healthy eating. Doctors perceived this was attributed to lack of breaks, inadequate canteen food selection, and canteen opening times.

Overall, doctors were dissatisfied with hospital canteen provision; satisfaction scores varying between the five hospital units. A majority viewed a healthy diet as an important influence over their work performance. Respondents' dissatisfaction with the provision of healthy and vegetarian options suggests that canteen provision does not reflect doctors' views or nutritional expectations. Most NHS hospital canteens are shared by doctors and hospital visitors, with hospital kitchens also providing catering for patients. It is therefore possible that poor canteen provision and dissatisfaction with canteen services may have a wider impact and relevance than to doctors alone. This study also suggests that NHS hospitals fail to cater for staff working on a 24-hour rota, with poor or absent evening and weekend catering provision.

A large proportion of doctors used the canteen on a weekly basis, with main meals and sandwiches accounting for the majority of purchases. Canteen use was higher in younger age groups and more junior job grades; this may be for a number of reasons including convenience.

Doctors reported reasonably healthy behaviours in relation to alcohol consumption, smoking, diet and exercise. However, daily water consumption, weekly breakfast consumption, and weekly aerobic exercise were low.

Even with the high reported levels of dissatisfaction surrounding healthy canteen provision, the proportion of doctors using such facilities remained high. More frequent canteen use was associated with higher health behaviour scores (i.e. unhealthy behaviours). The association between canteen use and health score was however weak, and must be interpreted cautiously.

Doctors reported relatively low negative and positive affect scores, suggesting low levels of psychological distress, depression, anxiety, and stress (low negative affect) but also low levels of pleasurable engagement with the environment (low positive affect). This study did not reveal any associations between affect and canteen use, satisfaction, or health score.

This study demonstrates that more frequent canteen use was associated with less healthy lifestyles and younger age. This survey accessed doctors of all job grades and specialties in two large NHS teaching Trusts and the results may be applicable to other large NHS teaching Trusts.

The generalisability of our results may be limited by the response rate of 43.7% with the resultant potential for responder bias. The sample under represents Senior House Officers and Specialist Registrars, who may have different work demands and health needs compared to their more senior consultant colleagues. Self reported data, as collected in this study, may be subject to social desirability bias, whereby reported perceptions and behaviours may not reflect true perceptions and behaviours, as well as recall bias.

The observed lack of association between affect and canteen use, satisfaction, or health scores may be attributable to untruthful reporting of affect by respondents for fear of identification or social desirability, or our failure to measure other potential influences such as stress.

In the absence of a validated health behaviour score, a score was developed from the Food Standards Agency questionnaire on healthy diet [17] with questions relating to alcohol, exercise, and smoking added. This questionnaire is an unweighted sum of a number of differently scored items and has not been subject to formal validation.

## Conclusion

This study demonstrates that many doctors do not consider their working environment to be conducive to healthy eating. Doctors perceived this to be caused by lack of breaks, lack of canteen food selection, and canteen opening times. These findings mirror those of the nurse based survey [9,10], and suggest that future research in this arena can view health care workers as one population as opposed to many job-specific subgroups.

Doctors' dissatisfaction with canteen facilities may be addressed through the provision of a greater number and variety of healthy eating options. In addition, improved canteen accessibility and availability of healthy options at evenings and weekends would cater for employees working a 24 hour rota.

While canteen provision and work environment may be important determinants of healthy eating, there may also be benefits in addressing doctors' lifestyle and promoting healthy choices [19]. Doctors should be encouraged to eat breakfast, take regular meal breaks, drink the recommended amount of water per day, and take regular aerobic exercise.

A multitude of factors interact to influence doctors' health at work; these factors include diet, workload, stress and mental health, and lifestyle [8,12-14]. A more comprehensive study into these factors would benefit the health of this large workforce and may ultimately lead to improved patient care [20], employee satisfaction, recruitment and retention of staff [15], and meeting governmental targets for improving the health of our nation [12]. Although doctors perceived canteen food to be unhealthy, its actual nutritional content was not determined in this study. Future work should look at the nutritional content of hospital canteen food, and determine whether NHS employers need to improve canteen provisions or alter employee perceptions.

## Competing interests

The authors declare that they have no competing interests.

## Authors' contributions

The idea for this study was conceived by JW and CJ. All authors contributed to the development of the design. JW undertook all data collection, analysis and wrote the first draft of the manuscript. All authors contributed to, and have approved, the final manuscript. SW was the primary supervisor of the study and is the guarantor.

## Supplementary Material

Additional File 1 Methods. Details of the study design and methods. Click here for file

Additional file 2 Table 3 – Canteen Use by Age, Gender and Job Grade. Additional results table. Click here for file

Additional file 3 Table 4 – One-way between-groups analysis of variance (ANOVA) of canteen use with age and job grade. Additional results table. Click here for file
