# Supplementary material for: Barriers to healthy eating by National Health Service (NHS) hospital doctors in the hospital setting: results of a cross-sectional survey
Source: BMC Res Notes. 2008 Aug 28;1:69. doi: 10.1186/1756-0500-1-69 (PMC2551607; doi:10.1186/1756-0500-1-69)
Supplement: Additional File 1 — Methods. Details of the study design and methods. [file 1756-0500-1-69-S1.doc]

METHODS

A confidential questionnaire, piloted to check for clarity of content, was distributed in March 2006 via the internal Trust postal systems. Mailing was undertaken by the Trust’s R&D department and completed questionnaires returned to their secure offices. One reminder was sent to all eligible doctors two weeks after the initial mailing. Additional questionnaires were placed in the junior doctors’ mess and at junior doctor teaching sessions at the University Hospital Birmingham.

*Participants:* The sampling frame was a list of doctors obtained via the R&D department in each Trust. All doctors working in Units with an on-site hospital canteen were eligible.

*Questionnaire:* The structured questionnaire asked about average weekly use and satisfaction with the hospital canteen,[1-2] perceived barriers to healthy eating,[1-2] lifestyle and dietary habits,[3] a visual analogue scale indicated health state today,[4] gender, age, height, weight, and job details. Negative mood and stress are associated with patterns of unhealthy eating.[5-7] The 10-item Positive and Negative Affect Schedule (PANAS)[8] was used to measure the potential influence of affect on doctors’ food preference and eating habits. The PANAS scale was selected because it was an easy to complete, well validated, measure of affect,[8] and has been used in previous studies of eating behaviours.[6] Normative data for the general adult UK population are available [9] and provide reference a mean positive affect score of 32 (32 males, 31 females) and negative affect score of 14 (males 14, females 15). A four point Likert scale measured satisfaction with the cost of goods, canteen location, opening times, selection of food and drink, availability of healthy options, and appearance/environment of the canteen. Responses were scored as three (very satisfied) to zero (not at all satisfied), and then summed to give each respondent a canteen satisfaction score (18 for very satisfied, 0 for not at all satisfied).

A health behaviour score (0 for extremely healthy behaviour to 25 for extremely unhealthy behaviour) was calculated from 11 questions on lifestyle and diet, adapted from the Food Standards Agency questionnaire on healthy diet.[3] These included questions on smoking status (score: non smoker=0, smoker=2), alcohol consumption in units (women’s scores: <13 = 0, 14 to 21 =1, 22 to 39 = 2, >40 = 3, men’s scores: <20 = 0, 21 to 28 = 1, 29 to 39 = 2, >40 = 3), separate episodes of aerobic exercise per week, frequency of weekly breakfast consumption (five point Likert scale, scored: 0=everyday, 4=never), food eaten for breakfast, snacking habits, daily fruit consumption, salt and takeaway consumption, number of glasses of water consumed daily, and drink choice when thirsty.

The perceived barriers to healthy eating were subdivided into three categories; job related factors included shift length, shift pattern, and lack of breaks; factors related to canteen provision included cost of goods, opening times, and lack of selection; personal factors included lack of motivation to eat healthily, lack of nutritional knowledge, and work related stress.

*Analysis:* Microsoft Access was used for data entry and SPSS 13.0 for data analysis. Means and standard deviations were calculated for continuous variables and frequency distributions for categorical variables.

Associations between the dependent variable health score and the covariates of gender and job grade were calculated using independent-samples t-test and one-way between-groups analysis of variance (ANOVA) respectively. Associations between health score and the covariates of canteen use, satisfaction score, positive and negative affect scores, age, and BMI were examined with Pearson product-moment correlation coefficients. Identical tests were repeated using satisfaction score, positive and negative affect scores, and canteen use as the dependent variables.

The results of parametric tests are reported for all analyses. Kolmogorov-Smirnov and Shapiro-Wilk tests demonstrated that the continuous data violated the assumption of normality. Non-parametric tests, including Spearman rank order correlation, Mann-Whitney U test, and Kruskal Wallis test, confirmed the outcomes of the parametric analyses.

**References**

1. Faugier J et al. Barriers to healthy eating in the nursing profession: Part 1. Nurs Stand. 2001. May 23 2001. 15(36): 33-36
2. Faugier J et al. Barriers to healthy eating in the nursing profession: Part 2. Nurs Stand. 2001. May 30 2001. 15(37): 33-35
3. Food Standards Agency. Are You a Healthy Eater? Source - [www.eatwell.gov.uk/agesandstages/teens/quiz2. Accessed 21 Jan 2006](http://www.eatwell.gov.uk/agesandstages/teens/quiz2. Accessed 21 Jan 2006)
4. EuroQol Group. EuroQol – a new facility for the measurement of health-related quality of life. Health Policy. 1990. 16:199–208
5. Ottley C. Food and Mood. Nurs Stand. 27 September 2000. 15(2): 46-52
6. Wegner KE, Smyth JM, Crosby RD, Wittrock D, Wonderlich SA, Mitchell JE. An evaluation of the relationship between mood and binge eating in the natural environment using ecological momentary assessment. Int J Eat Disord. Nov 2002. 32(3): 352-61.
7. Oliver G, Wardle J. Perceived effects of stress on food choice. Physiol Behav. 1999. 66(3): 511–515
8. Watson D, Clark LA. Development and Validation of Brief Measures of Positive and Negative Affect: The PANAS Scales. J Pers Soc Psychol. 1988. 54(6): 1063-1070
9. John R. Crawford JR, Henry JD. The Positive and Negative Affect Schedule (PANAS): Construct validity, measurement properties and normative data in a large non-clinical sample. British Journal of Clinical Psychology (2004), 43, 245–265
