# Supplementary material for: Barriers to healthy eating by National Health Service (NHS) hospital doctors in the hospital setting: results of a cross-sectional survey
Source: BMC Res Notes. 2008 Aug 28;1:69. doi: 10.1186/1756-0500-1-69 (PMC2551607; doi:10.1186/1756-0500-1-69)
Supplement: Additional file 2 — Table 3 – Canteen Use by Age, Gender and Job Grade. Additional results table. [file 1756-0500-1-69-S2.doc]

Table 3 – Canteen Use by Age, Gender and Job Grade

|  | |  | Doctors Using Canteen Facilities | | Average Canteen Use |  |  |
| --- | --- | --- | --- | --- | --- | --- | --- |
|  | | n | n | % |  | SD* | Range |
| Overall canteen use | | 328 | 229 | 69.8 | 2.07 | 2.09 | 0-10 |
| Canteen food and drink purchased | | 328 | 219 | 66.8 | 2.05 | 2.17 | 0-10 |
| Canteen main meals purchased | | 328 | 193 | 58.8 | 1.64 | 1.93 | 0-10 |
| Canteen use for other purposes (e.g. relaxation, meeting people) | | 328 | 24 | 7.3 | 0.11 | 0.57 | 0-8 |
| Age (years) | < 35 | 313 | 105 | 33.5 | 2.72 | 2.05 | 0-10 |
|  | 35 - 45 | 313 | 57 | 18.2 | 1.77 | 1.93 | 0-9 |
|  | > 45 | 313 | 63 | 20.1 | 1.68 | 2.04 | 0-10 |
| Gender | Male | 315 | 132 | 41.9 | 2.10 | 2.09 |  |
|  | Female | 315 | 94 | 29.8 | 2.15 | 2.04 |  |
| Job Grade | Foundation Year 1 (FY1) | 24 | 22 | 91.7 | 3.26 | 2.68 | 0-10 |
|  | Senior House Officer (SHO) | 49 | 42 | 85.7 | 2.93 | 1.87 | 0-7 |
|  | Specialist Registrar | 68 | 55 | 80.1 | 2.44 | 1.98 | 0-8 |
|  | Associate Specialist | 9 | 5 | 55.6 | 1.11 | 1.34 | 0-3 |
|  | Staff Grade | 11 | 7 | 63.6 | 2.00 | 2.14 | 0-5 |
|  | Consultant | 151 | 93 | 61.6 | 1.62 | 1.93 | 0-10 |

* Standard Deviation
