# Supplementary material for: Barriers to healthy eating by National Health Service (NHS) hospital doctors in the hospital setting: results of a cross-sectional survey
Source: BMC Res Notes. 2008 Aug 28;1:69. doi: 10.1186/1756-0500-1-69 (PMC2551607; doi:10.1186/1756-0500-1-69)
Supplement: Additional file 3 — Table 4 – One-way between-groups analysis of variance (ANOVA) of canteen use with age and job grade. Additional results table. [file 1756-0500-1-69-S3.doc]

Table 4 One-way between-groups analysis of variance (ANOVA) of canteen use with age and job grade

| Doctors Canteen use compared by Job Grade (Gabriel Post Hoc Analysis) | | Mean difference | SE* | p | 95% CI |
| --- | --- | --- | --- | --- | --- |
| FY1  Compared to: | SHO | 0.332 | 0.49 | 1.00 | -1.11 to 1.77 |
|  | Specialist Registrar (SpR) | 0.819 | 0.47 | 0.69 | -0.54 to 2.17 |
|  | Associate Specialist | 2.15 | 0.78 | 0.071 | -0.088 to 4.39 |
|  | Staff Grade | 1.26 | 0.73 | 0.70 | -0.84 to 3.37 |
|  | Consultant | 1.64 | 0.44 | 0.001 | 0.45 to 2.83 |
|  |  |  |  |  |  |
| SHO  Compared to: | SpR | 0.49 | 0.37 | 0.96 | -0.61 to 1.59 |
|  | Associate Specialist | 1.82 | 0.72 | 0.10 | -0.16 to 3.80 |
|  | Staff Grade | 0.93 | 0.67 | 0.89 | -0.92 to 2.78 |
|  | Consultant | 1.31 | 0.33 | 0.001 | 0.37 to 2.24 |
| Doctors Canteen use compared by Age (Tukey Post Hoc Analysis) | |  |  |  |  |
| Age <35 Compared to: | Age 35-45 | 0.95 | 0.28 | 0.002 | 0.29 to 1.61 |
|  | Age >45 | 1.04 | 0.27 | 0.000 | 0.40 to 1.68 |

* Standard Error
